# Improvement of Mesenchymal Stromal Cell Proliferation and Differentiation *via* Decellularized Extracellular Matrix on Substrates With a Range of Surface Chemistries

**DOI:** 10.3389/fmedt.2022.834123

**Published:** 2022-03-17

**Authors:** Michael C. Yang, Andrea J. O'Connor, Bill Kalionis, Daniel E. Heath

**Affiliations:** ^1^Department of Biomedical Engineering, University of Melbourne, Parkville, VIC, Australia; ^2^Department of Maternal-Fetal Medicine, Pregnancy Research Centre, Royal Women's Hospital, Parkville, VIC, Australia; ^3^Department of Obstetrics and Gynecology, University of Melbourne, Parkville, VIC, Australia

**Keywords:** decellularized extracellular matrix, cell secreted matrix, mesenchymal stem cell, mesenchymal stromal cell, biomaterial, surface chemistry, stem cell manufacturing, tissue engineering

## Abstract

Decellularized extracellular matrix (dECM) deposited by mesenchymal stromal cells (MSCs) has emerged as a promising substrate for improved expansion of MSCs. To date, essentially all studies that have produced dECM for MSC expansion have done so on tissue culture plastic or glass. However, substrate surface chemistry has a profound impact on the adsorption of proteins that mediate cell-material interactions, and different surface chemistries can cause changes in cell behavior, ECM deposition, and the *in vivo* response to a material. This study tested the hypothesis that substrate surface chemistry impacts the deposition of ECM and its subsequent bioactivity. This hypothesis was tested by producing glass surfaces with various surface chemistries (amine, carboxylic acid, propyl, and octyl groups) using silane chemistry. ECM was deposited by an immortalized MSC line, decellularized, and characterized through SDS-PAGE and immunofluorescence microscopy. No significant difference was observed in dECM composition or microarchitecture on the different surfaces. The decellularized surfaces were seeded with primary MSCs and their proliferation and differentiation were assessed. The presence of dECM improved the proliferation of primary MSCs by ~100% in comparison to surface chemistry controls. Additionally, the adipogenesis increased by 50–90% on all dECM surfaces in comparison to surface chemistry controls, and the osteogenesis increased by ~50% on the octyl-modified surfaces when dECM was present. However, no statistically significant differences were observed within the set of dECM surfaces or control surfaces. These results support the null hypothesis, meaning surface chemistry (over the range tested in this work) is not a key regulator of the composition or bioactivity of MSC-derived dECM. These results are significant because they provide an important insight into regenerative engineering technologies. Specifically, the utilization of dECM in stem cell manufacturing and tissue engineering applications would require the dECM to be produced on a wide variety of substrates. This work indicates that it can be produced on materials with a range of surface chemistries without undesired changes in the bioactivity of the dECM.

## Introduction

Over 700 clinical trials have assessed the therapeutic potential of mesenchymal stromal cells (MSCs) to treat pathologies as diverse as Crohn's disease, graft-vs.-host disease, and multiple sclerosis (1). However, the low *in vivo* prevalence of MSCs necessitates prolonged *ex vivo* expansion to produce sufficiently large quantities of MSCs for clinical therapies (2, 3), but key MSC properties including proliferative capacity and differentiation potential are rapidly lost when MSCs are expanded using conventional *ex vivo* cell culture technologies such as tissue culture flasks (3–5). When expanded under these conditions, a large proportion of the population are “filler cells” that no longer exhibit the desired MSC phenotype. This loss of phenotype limits the clinical success of MSC therapies and illustrates the need to develop new cell culture strategies that enable the large-scale expansion of highly potent MSCs.

Decellularized extracellular matrix (dECM) isolated following *in vitro* culture of MSCs is one of the newer and more promising techniques for production of large numbers of viable MSCs. We recently reviewed how MSCs cultured on dECM surfaces maintain key phenotypic properties during prolonged expansion when compared with traditional cell culture surfaces including untreated tissue culture plastic, monolayers of adsorbed proteins, and Matrigel (6, 7). For example, MSCs grown on these dECMs show a 425-fold increase in colony forming unit capacity over 8 passages *in vitro* (8), and these cells resulted in a 5-fold increase in bone formation capacity in an *in vivo* ectopic bone assay (9). Remarkably, dECM can rejuvenate key properties of less potent MSCs collected from aged donors or those that have lost potency due to prolonged *ex vivo* culture on tissue culture plastic (10, 11). The beneficial properties of dECM materials are thought to arise from their biological complexity and ability to better mimic key features of the MSC niche (12). Specifically, cells cultured on dECM preferentially express proliferation genes compared to matrix producing genes on tissue culture plastic (13). Moreover, MSCs cultured on dECM materials produce significantly lower levels of reactive oxygen species and maintain telomerase activity, which may delay the onset of senescence (6). While dECM is markedly better than the majority of synthetic cell culture substrates, there is further research to be done to improve the bioactivity and scalability of these dECM biomaterials.

Conventionally, dECM is produced by low passage number (< P5) primary MSCs (2, 5), limiting the scalability of the technique. However, we showed that stably transfected MSC cell lines can be used to produce orders of magnitude (~10^6^) more high quality dECM in comparison to primary cells. Moreover, these dECMs were produced from a single cell source, eliminating the patient-to-patient variability from primary MSCs (14). A subsequent study improved the scalability of these cell line-derived matrices by showing that biologically active molecules in the matrices could be solubilized and transferred to coat ~1.3–5.2 times the surface area of the native dECM while retaining some of the key properties of the native dECM (15). These advances increase the technological feasibility of generating dECM for the expansion of MSCs, toward clinical scale production.

To the authors' knowledge, all studies to date that produced dECM for MSC expansion have done so on tissue culture plastic or glass. However, substrate properties can impact the response of a cell to a material. One particularly important physical property is substrate surface chemistry. Unless functionalized with bioactive motifs, cells rarely interact directly with a biomaterial. Instead, the interactions are governed by a layer of proteins and biomolecules that adsorb to the material surface (16, 17). Controlling the surface chemistry influences the amount of adsorbed protein, the composition of the protein layer, the orientation of adsorbed proteins, and the degree of protein denaturing (18–21). For example, hydrophilic and electrically neutral surfaces like PEG or zwitterionic materials hold onto their waters of hydration, preventing the adsorption of biomolecules, and thus these surfaces are largely not adherent to cells (22–24). Additionally, surfaces that are more hydrophobic tend to be more proinflammatory *in vivo* due to greater denaturation of proteins upon adsorption (25). Consequently, manipulation of surface chemistry indirectly influences the cellular response (26, 27).

Despite significant work on how a biomaterial's surface chemistry impacts its interaction with cells, its impact on the deposition and bioactivity of MSC dECM has not been well explored. Previous investigations have centered around fabrication of a specific biomaterial and subsequent dECM protein binding onto this scaffold (28, 29), rather than targeted manipulation of surface chemistry and its ensuing effects on MSC dECM. This is an important research gap because there is potential for dECM to be produced on a wide variety of different biomaterials with varying surface chemistries for either tissue engineering or cell expansion applications. We hypothesize that surface chemistry during dECM deposition modifies the bioactivity of the dECM, and this will impact the proliferation and differentiation of primary MSCs cultured on the dECM. This hypothesis was tested by functionalizing glass coverslips with silanes to possess hydrophilic (carboxylic acid or amine) groups or hydrophobic (propyl or octyl) groups. The surfaces were used as cell culture substrates for dECM deposition, and the proliferation and differentiation of primary MSCs was assessed. The presence of dECM improved the proliferation and adipogenesis of MSCs in comparison to surface chemistry controls and improved osteogenesis on the octyl-modified surfaces. Surprisingly, we did not observe significant differences when comparing the proliferation or differentiation of MSCs on the dECM surfaces. These results indicate that dECM coatings can be produced with minimal variations in properties over the range of surface chemistries assessed in this work. These results are significant because they facilitate the use of dECM technology on a wide range of materials without significant changes to the bioactivity of the dECM.

## Materials and Methods

### Materials

3-aminopropyl triethoxysilane, trimethoxypropylsilane, trimethoxyoctylsilane, and propylamine solutions were purchased from Sigma Aldrich (Castle Hill, NSW, Australia). Carboxyethylsilanetriol was obtained as a 25% solution obtained from Gelest (Morrisville, PA, USA). 3-aminopropyl triethoxysilane was purchased as a 99% solution and carboxyethylsilanetriol was obtained as a 25% solution.

### Surface Modification Using Silane Chemistry

All silanization reactions were performed in liquid phase. 3-aminopropyl triethoxysilane, trimethoxypropylsilane, and trimethoxyoctylsilane were separately diluted to concentrations of 1% v/v in 95% ethanol/5% water. 0.5% propylamine was used as a catalyst for during silanization with 3-aminopropyl triethoxysilane. Carboxylethylsilanetriol was diluted to 1% in 0.1 M acetic acid. Glass coverslips were first cleaned with oxygen plasma and placed in the freshly made silane solutions for 4 h at room temperature. After silanization, the coverslips were rinsed with respective diluents and dried under a stream of nitrogen. The coverslips were stored for no longer than 24 h before use.

### Surface Characterization of Glass Substrates

Water contact angle was measured using a Dataphysics OCA 20 system. Silanized and control coverslips were washed with ethanol and deionized water and dried under a stream of nitrogen. The static water contact angle was measured by using a glass syringe to place a ~8 μL water droplet on the glass coverslips. Measurements were taken at room temperature. The contact angle of each droplet was measured through the denser water phase.

The surface topography of silanized and control coverslips was assessed with atomic force microscopy (AFM). The silanized glass coverslips were stored under nitrogen until AFM measurements were taken. A Tap 300 cantilever (Budget Sensors) was used with a resonant frequency of 300 kHz, and a spring constant of 40 N/m. Scans were taken over a 5 × 5 μm area.

The zeta potential of the glass substrates was measured using an Anton Paar SurPASS analyzer. Coverslips were diced to 20 × 10 mm rectangles and silanized as previously described. Measurements were taken over an aqueous HCl titration from pH 3–11 at a pressure of 400 mbar, with 0.001 M aqueous KCl used as the electrolyte, with at least 12 data points collected for each sample at a temperature of 21°C.

### dECM Production and Primary Cell Culture

The decellularized extracellular matrices were produced using the immortalized DMSC23 cell line as we described previously (14). Briefly, cells were seeded at 1,000 cells/cm^2^ onto silanized or control glass coverslips and cultured to confluence with media changes every 2–3 days. The cells were cultured in α-MEM basal medium (Sigma Aldrich) with 10% fetal bovine serum (Gibco), 100 U/mL penicillin/streptomycin, and 2 mM L-glutamine. Cells were maintained at 37^°^C and 5% CO_2_. At the onset of confluence, 50 mM L-ascorbate 2-phosphate was added to the medium to induce extracellular matrix deposition. After an additional 7 days of culture, cultures were decellularized using 20 mM ammonium hydroxide/0.5% Triton X-100 at 37°C for 5 min, then rinsed three times with PBS to remove residual detergent. Passages 18–30 of the immortalized DMSC23 cells were used for matrix deposition.

Human term placentae from uncomplicated pregnancies were used to isolate primary MSCs from the *decidua basalis* according to our previously published protocol (1, 30–32). Written and informed patient consent and ethics approval from the Royal Women's Hospital Human Research and Ethics Committee was obtained (HREC #14/35). The cells were cultured in α-MEM basal medium (Sigma Aldrich) with 10% fetal bovine serum (Gibco), 100 U/mL penicillin/streptomycin, and 2 mM L-glutamine. Cells were maintained at 37°C and 5% CO_2_. Medium was changed every 2–3 days, with passaging when cells reached ~70% confluence. Primary MSCs used in this work were a pooled sample of cells from 5 donors. Passages 4–5 were used in this study.

### Confocal Microscopy of Stained ECM

Samples were fixed in 10% formalin prior to staining. dECM microarchitecture was visualized by staining for collagen I as previously described (14, 15) using a 1/1,000 v/v dilution of mouse anti-collagen I primary antibody (Sigma Aldrich lot #067M4805V) and 2 μg/mL donkey anti-mouse Alexa Fluor 488 (Invitrogen) secondary antibody.

### SDS-PAGE

SDS-PAGE was used to compare the compositions of the dECMs. Protein extracts were prepared using the Pierce Protein Assay Kit according to manufacturer instructions and resuspended in 7.5 μL XT sample buffer/1.5 μL XT reducing agent (Bio-Rad), placed in a 95°C heating block for 10 min, and centrifuged for 2 min at 14,000 g. Samples were loaded into 1.0 mm 4–12% gradient Bis-Tris Criterion XT Precast Gel (Bio-Rad), with collagen I (Sigma Aldrich) and Precision Plus protein ladder (Bio-Rad) used as controls. Loaded gels were placed into XT MOPS buffer (Bio-Rad) and electrophoresed at 150 V for ~100 min. Gels were then stained with BioSafe Coomassie (Bio-Rad) for at least 2 h and washed with deionized water three times for 5 min each. Bands were visualized using a GE Image Scanner III, with images taken using ImageQuant TL software (GE Healthcare).

### Cell Proliferation Assay

Primary *decidua basalis* MSCs were seeded onto dECM and control surfaces at a density of 2,000 cells/cm^2^. Cell surface densities were determined using a PicoGreen DNA quantification assay at 7 days according to our previously published protocol (33). Briefly, cultures were rinsed three times with PBS, cells were lifted using trypsin-EDTA, suspensions were centrifuged at 230 g for 5 min to form a cell pellet, and cells were resuspended in 1 mL cysteine buffer (5 mM cysteine-HCl, 5 mM Na_2_EDTA in PBS). The cells were lysed by mixing 150 μL cell suspension with 150 μL 0.25 mg/mL papain (Sigma Aldrich) and incubated for 20 h at 60°C. Lysed cell solution (50 μL) was transferred to a well of a black 96-well plate, and 50 μL of 1:200 PicoGreen solution (Thermo Fisher Scientific) in TE buffer (10 mM Tris-HCl, 1 mM EDTA, pH 7.5) was added to the well. The plate was incubated at room temperature in the dark for 5 min. The fluorescence of each well was measured at an excitation wavelength of 480 nm and emission of 520 nm. The number of cells were determined using a calibration curve generated from a known number of cells, and the cell surface density (cells/area) were calculated by normalizing that the number of cells to the cell culture surface area. Experiments were performed with a minimum of three replicates.

### Adipogenic Differentiation of Primary MSCs

Adipogenesis assays were performed accordingly to our previously established protocol (30), with changes made to the seeding density of the primary *decidua basalis* MSCs. Briefly, primary MSCs were seeded at a density of 5,000 cells/cm^2^ onto dECM and control surfaces and cultured in α-MEM supplemented with StemXVivo Adipogenic Supplements (R&D Systems) according to the manufacturer's instructions. Media was changed every 3–4 days. At the end of the 21-day culture period, cultured were fixed in 10% formalin, and adipogenesis was assessed through the staining of lipid droplets using a solution of Oil Red O (36 mg in 20 mL 70% isopropanol). Stained cultures were visualized using brightfield microscopy. Quantification of triglyceride accumulation was performed by solubilizing stained cells with 100% isopropanol for 10 min. The optical density of the solution was then measured at a wavelength of 520 nm using a SpectraMax Plus microplate reader (Molecular Devices).

### Osteogenic Differentiation of Primary MSCs

Osteogenesis assays were performed as previously described (1, 30). Briefly, primary *decidua basalis* MSCs were seeded at a density of 4,200 cells/cm^2^ onto dECM and control surfaces and cultured in α-MEM supplemented with StemXVivo Osteogenic Supplements (R&D Systems) according to the manufacturer's instructions. Medium was changed every 3–4 days. Osteogenesis was quantified using an OsteoImage mineralization assay (Lonza) according to the manufacturer's protocol. Briefly, cells were washed with PBS and fixed using ethanol, washed with diluted Wash Buffer, incubated with Staining Reagent for 30 min, washed 3x with Wash Buffer, and measured using a Fluostar Optima.

### Statistical Analysis

Quantitative data is given as mean ± standard deviation. Minitab software was used to perform two-way ANOVA with Tukey's *post-hoc* statistics tests, with *p*-values of ^*^ <0.05, ^**^ <0.01, and ^***^ <0.001 considered significant. Cell culture experiments were performed with a minimum of three replicates.

## Results

### Substrates With Various Surface Chemistries Can Be Generated Through Silanization of Glass Coverslips

Silane chemistry is a well-established method of functionalizing glass surfaces with desired chemical moieties. In this work, silanes were used to control the surface chemistry of glass substrates to assess how surface chemistry impacted dECM deposition and bioactivity. Specifically, glass coverslips were modified with one of four silane species. Two silanes were selected to produce hydrophilic surfaces (functionalization with charged amine or carboxylic acid groups) at physiological pH, while the other two produced relatively hydrophobic surfaces (functionalization with linear propyl or octyl groups). Unmodified glass coverslips were used as controls. Silanization was chosen as the method of controlling surface chemistry instead of using a range of different substrate materials to better control other material parameters such as stiffness and creep.

**Figure 1:**
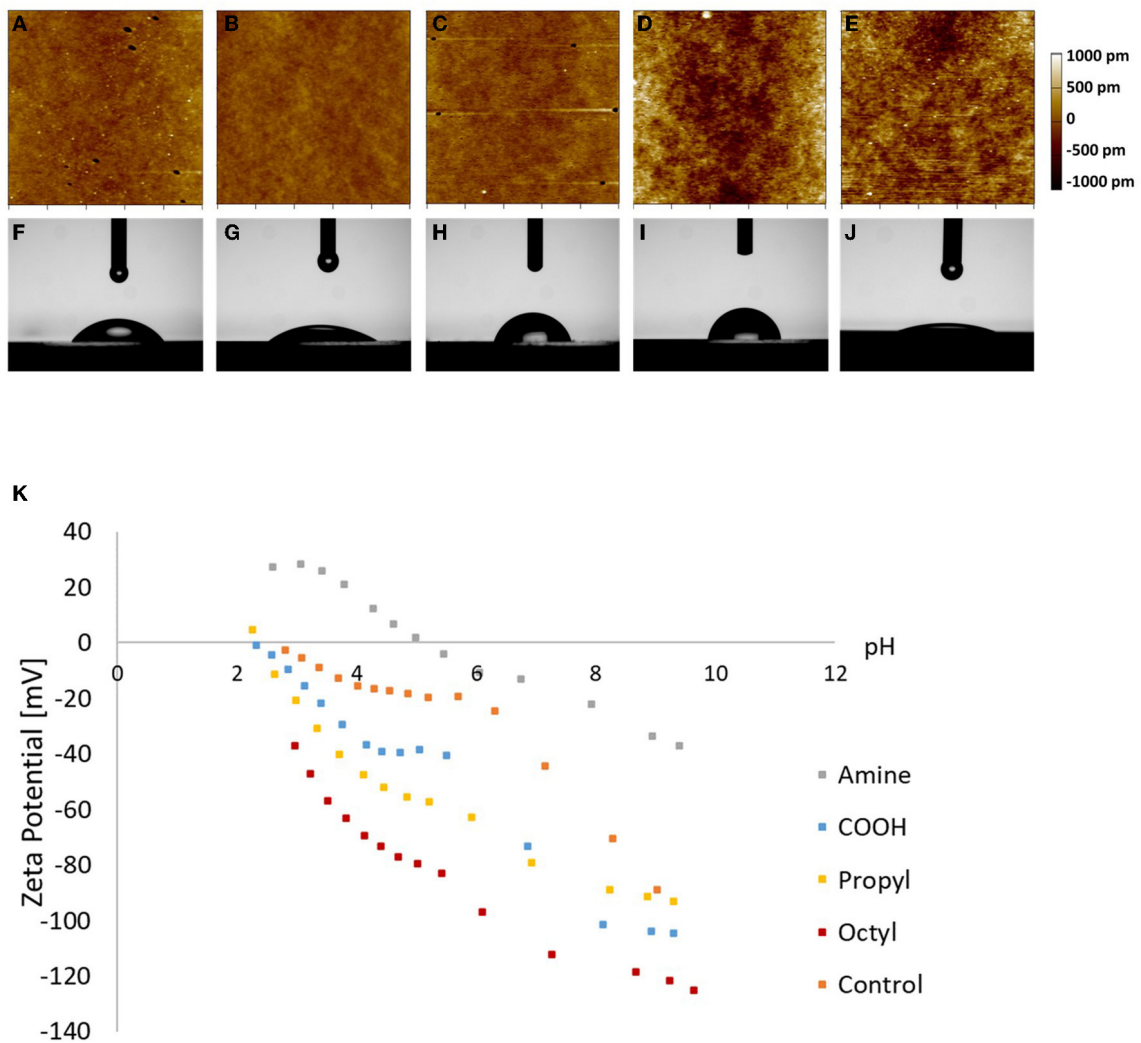
Characterization of **(A–E)** surface roughness, **(F–J)** water contact angle, and **(K)** zeta potential of **(A,F)** amine-, **(B,G)** carboxylic acid-, **(C,H)** propyl-, and **(D,I)** octyl-modified glass surfaces, and **(E,J)** glass control.

The surface roughness, water contact angle, and zeta potential for the various surfaces were characterized (Figure 1). Silanization can cause changes in surface roughness, and MSCs have been shown to alter their behavior depending on surface roughness (34–38). Therefore, it was necessary to experimentally determine the impact of silanization on the surface roughness of the substrates. Resulting AFM maps are seen in Figure 1. Visually, octyl-modified surfaces appear rougher than the other surfaces. This is corroborated by quantitative AFM results presented in Table 1; however, the quantification illustrates that the magnitude of change in the surface roughness is small, indicating relative homogeneity between groups.

**Table 1:** Surface roughness, water contact angle, and surface charge at physiological pH of silane-modified surfaces.

**Surface**	**Average roughness** **(pm)**	**Contact** **angle** **(°)**	**Estimate of zeta** **potential at physiological pH^†^** **(mV)**
Amine	268 ± 42	55 ± 1	−17.9 ± 0.8
Carboxylic acid (COOH)	278 ± 26	33 ± 1	−83.8 ± 2.2
Propyl	283 ± 22	76 ± 3	−81.5 ± 1.1
Octyl	350 ± 124	81 ± 5	−112.0 ± 0.4
Glass	278 ± 56	11 ± 1	−47.2 ± 1.2

† *Values were determined by evaluating best fit lines at a pH of 7.4*.

To verify that the surface hydrophilicity and chemistry of the glass coverslips were modified, the water contact angle and zeta potential were measured (Figure 1; Table 1). Unmodified glass surfaces had the greatest hydrophilicity, with water droplets having contact angles of 11 ± 1°. In contrast, the prepared surfaces had increased water contact angles, illustrating that the silanization treatment altered the surface chemistry. Additionally, surfaces modified with the amine- and carboxylic acid-bearing silanes were more hydrophilic as observed through lower contact angles (55 ± 1 and 33 ± 1°, respectively) than the propyl- and octyl-bearing silanes (76 ± 3 and 81 ± 5°, respectively), illustrating that the hydrophilicity of the surfaces could be tailored in the desired manner. Interestingly, contact angle for the propyl- and octyl-modified surfaces was still <90°, indicating that the surfaces were still relatively hydrophilic.

The zeta potential of the surfaces was assessed, as seen in Figure 1. Amine-modified samples had a higher isoelectric point compared to carboxylic acid-modified surfaces, as expected, and the trends in the zeta potential data are consistent with previous literature on other surface modification techniques such as self-assembled monolayers on gold (39). Similarly, the negative charge observed on the hydrophobic surfaces is also consistent with previous reports. This is often attributed to preferential adsorption of OH^−^ ions at the interface; however, there is still debate on the exact mechanism (40–43). These results illustrate that the silanization alters surface chemistry, resulting in distinct electrostatic environments for each treatment. These changes in zeta potential could impact protein adsorption, which could then impact cell attachment and subsequent dECM production.

### Substrate Surface Chemistry Does Not Significantly Alter dECM Composition or Microarchitecture

**Figure 2:**
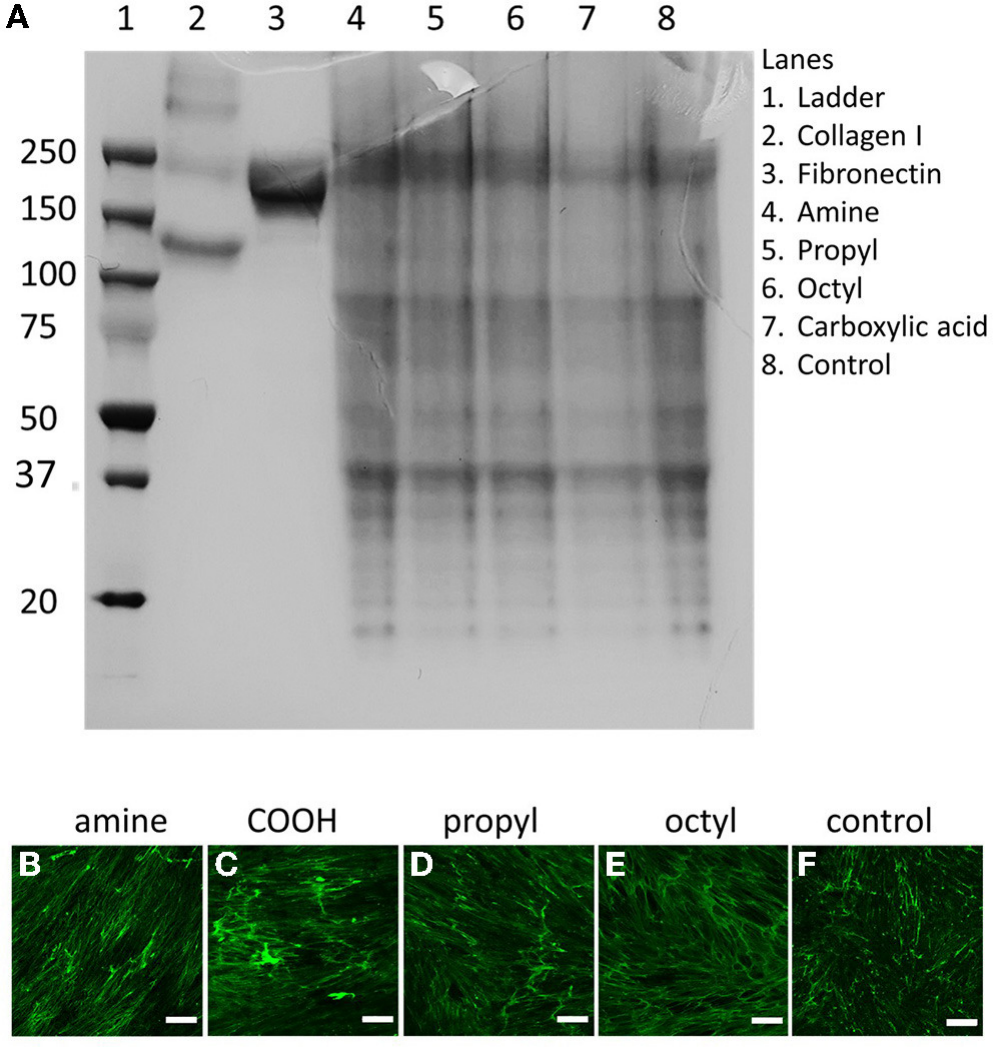
**(A)** SDS-PAGE and **(B–F)** confocal microscopy images of dECMs on surfaces with different surface chemistries surface chemistries. Matrices deposited on **(B)** amine-, **(C)** carboxylic acid-, **(D)** propyl-, and **(E)** octyl-modified surfaces are stained for collagen I, compared to **(F)** unmodified controls. Scale bars represent 200 μm.

dECM was produced by the DMSC23 cell line as we have previously reported, and the matrices produced on the various surfaces were assessed by SDS-PAGE and fluorescence microscopy to look for changes in composition and microstructure (Figure 2). SDS-PAGE produced multiple bands, illustrating that the dECMs were a complex mixture of biomolecules. Interestingly, there was no major differences in the banding pattern between samples, indicating that the dECM had a similar composition across all surfaces. Similarly, confocal microscopy showed the presence of matrix on all samples but there was no visually discernible difference between ECMs, suggesting that surface chemistry does not play a significant role in determining structure of secreted ECM.

### Surface Chemistry Did Not Significantly Alter the Proliferation of MSCs on dECM or Control Surfaces

**Figure 3:**
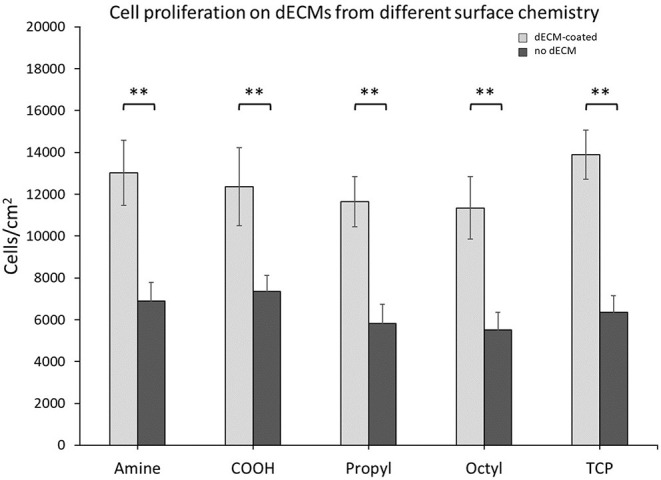
Cell proliferation on dECMs and surface chemistry control surfaces. Brackets indicate significant differences, with ** <0.01. All means were statistically compared using a two-way ANOVA and a Tukey's *post-hoc* test. However, only differences between a dECM-coated surface and its surface chemistry control are indicated for clarity.

Primary MSCs were seeded onto dECMs or surface chemistry control surfaces, and the cell number was assessed after 7 days (Figure 3). dECM improved primary MSC proliferation by a factor of ~2 when compared to surface chemistry controls. These results are consistent with our previous work (11, 14). Interestingly, no differences in proliferation were observed between the different dECM treatments or within the surface chemistry control treatments. These results indicate that the range of surface chemistries explored in this work do not alter the bioactivity of MSC dECM for MSC proliferation.

### Surface Chemistry Did Not Significantly Alter the Adipogenic Potential of MSCs on dECM or Control Surfaces

**Figure 4:**
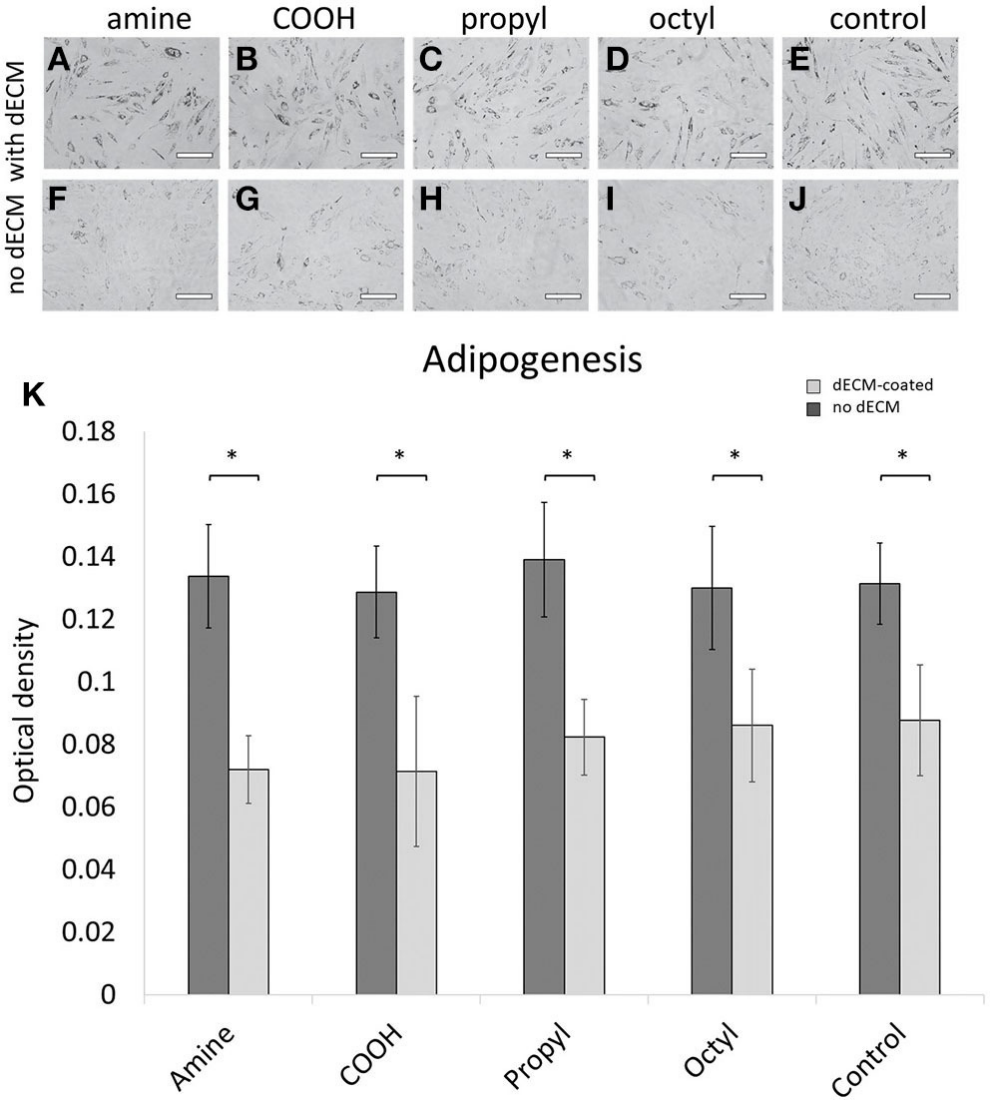
Primary MSCs were differentiated down adipogenic lineages and stained for adipogenesis using Oil Red O on **(A–E)** dECM-coated surfaces and **(F–J)** surface-modified glass controls. **(K)** Adipogenesis was quantified through solubilization and intensity measurements of Oil Red O stain. Scale bars represent 200 μm. Brackets indicate significant differences, with ^*^ <0.05. All means were statistically compared using a two-way ANOVA and a Tukey's *post-hoc* test. However, only differences between a dECM-coated surface and its surface chemistry control are indicated for clarity.

Primary MSCs were cultured on the dECM and control surfaces with adipogenic supplements for 21 days. After the induction period, Oil Red O revealed the presence of lipid droplets inside the cells (Figure 4). Qualitatively, MSCs exhibited greater adipogenic differentiation on dECMs in comparison to their surface chemistry control surface as observed through greater concentrated intracellular lipid droplets. Cells on control surfaces still displayed adipogenic differentiation, although lipid droplets were more sparsely distributed. Quantitatively, cells cultured on the dECM surfaces exhibited a statistically significant increase in adipogenesis, with an ~2-fold increase in the amount of triglyceride accumulation compared to those cultured on surface chemistry controls. However, no significant differences were observed between adipogenesis on surfaces with dECMs or between surface chemistry control surfaces. These results indicate that surface chemistry, over the range of values studied, is not a significant regulator of adipogenesis, nor does it impact the bioactivity of the deposited dECM for adipogenic applications.

### Surface Chemistry Did Not Significantly Alter the Osteogenic Potential of MSCs on dECM or Control Surfaces

**Figure 5:**
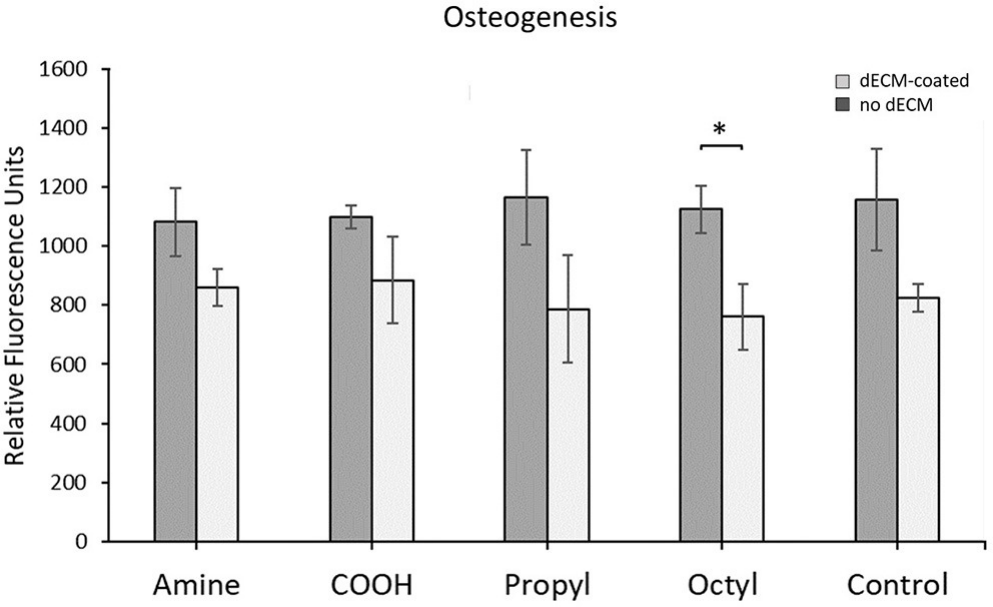
Quantification of MSC osteogenesis when cultured on silanized surfaces with and without dECM. Brackets indicate significant differences, with ^*^ <0.05. All means were statistically compared using a two-way ANOVA and a Tukey's *post-hoc* test. However, only differences between a dECM-coated surface and its surface chemistry control are indicated for clarity.

Primary MSCs were differentiated down the osteogenic lineage over 14 days, and differentiation was quantified as shown in Figure 5. As with the proliferation and adipogenesis data, no statistical difference was observed between any of the dECM surfaces or the control surfaces. However, we observed in increase in osteogenesis on the octyl-modified surface in the presence of dECM. These results indicate that surface chemistry is not a key factor in controlling the osteogenic potential of dECM over the range of surface chemistries assessed in this work, as no statistical differences were observed between the dECM groups.

## Discussion

Environmental parameters are key drivers of MSC behavior. Surface chemistry alters MSC differentiation and protein expression. For example, Lanniel et al. fabricated polyacrylamide hydrogels and modified them with amine, carboxyl, or phosphate functional groups. The researchers found that phosphate moieties resulted in increased expression of osteogenic marker Runx2, whereas myogenic marker MyoD1 was highest on surfaces with carboxylic acid moieties (26). While the specific surface functional groups and differentiation pathways differed from those studied in our investigation, they demonstrate the dependence of MSC differentiation behavior on surface chemistry. Additionally, Bachhuka et al. demonstrated that collagen deposition was different when fibroblasts were cultured on plasma polymer surfaces containing amine, carboxylic acid, and propyl groups in comparison to glass controls. Specifically, the researchers looked at the production of collagen type I and collagen type III across the surfaces. The researchers found that the amount of collagen type I and collagen type III was greatest on amine-bearing surfaces after 3 days of culture. However, all the surfaces had a similar amount and ratio of collagen type I and collagen type III after 16 days of culture (44).

Surface chemistry affects the behavior of adherent cells and impacts the composition of the ECM that they deposit. As such, it seems intuitive that surface chemistry would play an important role in regulating the bioactivity of MSC-derived dECM, but this has never been investigated. To address this research gap, we produced dECMs on glass surfaces modified with amine, carboxylic acid, propyl, or octyl functional groups through silane chemistry. The four silanes were chosen for multiple reasons. Cell culture substrates experience protein adsorption, and this adsorption facilitates cell adhesion and their subsequent behavior (19). The two hydrophobic surfaces were selected as hydrophobicity promotes the denaturing of proteins upon adsorption (22). In the short term, the effect of this denaturation is often the presence of fewer viable cells and reduced cell adhesion (27). However, over longer time periods, hydrophobic surfaces show comparable cell activity to hydrophilic surfaces, most likely due to the masking of surface properties by cells and extracellular matrix (45, 46). Additionally, the surface charge is a main driver which influences the species of proteins which adsorb to a surface (47). As such, creating surfaces which are functionalized with positive (amine) and negative (carboxylic acid) groups at physiological pH could influence protein adsorption and the subsequent bioactivity of deposited ECM. Different charged chemical moieties could have been selected to functionalize our surfaces; however, the amine and carboxylic acid groups were selected as they are commonly found in biological systems such as charged amino acids.

Our results illustrate that dECM can be used to improve proliferation and adipogenesis on the dECM surfaces compared to their surface chemistry controls and improve osteogenesis on octyl-modified surfaces. However, our results illustrate that substrate surface chemistry does not significantly impact the composition or bioactivity of MSC-derived dECM over the range of surface chemistries investigated, as no significant differences were observed in proliferation and differentiation results between the dECM surfaces. At first glance, these results seem to contrast those reported by Bachhuka et al. who observed changes in ECM composition with surface chemistry. However, they found that substrate surface chemistry only impacted the collagen type I and collagen type III deposition process over short timeframes (3 days), and all surfaces appeared similar by the end of the experiment on day 16 (44). The dECM substrates produced in this work take ~2 weeks to obtain. During the first week, the cells are grown to confluence, and during the second week the cells are supplemented with ascorbic acid to stimulate collagen production (48). We attribute the relative insensitivity of our dECMs to surface chemistry to two main reasons. First, we only assessed matrix composition after ~14 days. Our experiments simply may have skipped over the relatively short timepoints over which changes in matrix composition are observed. Second, the cultures were supplemented with ascorbic acid during the ECM deposition phase. This is a common practice as the supplementation has been shown to stimulate matrix deposition of ECM by many cell types *in vitro* including vascular smooth muscle cells, fibroblasts, and MSCs (49–51), enabling a larger amount of matrix to be produced in the same timeframe. Specifically, the ascorbic acid stimulates the deposition of collagen (48), and this may override surface chemistry affects, resulting in a dECM with a similar composition across all surfaces.

While the results of this study did not support the hypothesis that surface chemistry would be a key parameter that impacted the bioactivity of MSC dECM, these results are still significant to the field. To fully utilize the potential of MSC dECM in stem cell manufacturing and in tissue engineering applications, the dECM layers will need to be produced on a variety of different materials depending on the application. As such, it is critical to know that the dECM can be produced on surfaces with a range of surface chemistries, and that this will not significantly alter its biological properties. However, it must be acknowledged that the water contact angles of the surfaces studied in this work ranged from 11 to 81^°^ and looked at 5 different surface chemistries, including the glass control. Surfaces such as hydrophobic polymers with much greater hydrophobicities and surfaces with other charged moieties may produce different results. Additionally, silane layers on glass can undergo hydrolysis during prolonged exposure to an aqueous environment. While we have selected silanization conditions best suited for use in cell culture (52, 53), this may result in transience in the surface chemistry of the substrates during prolonged cell culture. However, it has been previously demonstrated that the contact angle of silane layers on glass can be relatively stable for hundreds of hours (54), while the key steps of protein adsorption and cell adhesion occurs in seconds to hours. Additional future work could include performing similar experiments on a variety of common biomedical surfaces with stable surface chemistries. However, the use of multiple substrates does not allow for control over other parameters that influence cell fate such as substrate stiffness and viscoelasticity, which is why this strategy was not pursued in this research.

## Conclusion

Here, we investigate the effect of controlling surface chemistry on the bioactivity of MSC-derived extracellular matrices. Extracellular matrices deposited over 2 weeks on surfaces with amine, carboxylic acid, propyl, and octyl functional moieties were able to improve proliferation and adipogenic differentiation on all surfaces and improve osteogenic differentiation on octyl-modified surfaces. However, no significant differences in proliferation or differentiation were observed when comparing dECM. This is an important finding as it illustrates that surface chemistry, over the range assessed in this work, is not a key variable that must be controlled during secretion of extracellular matrix by MSCs. Furthermore, these results indicate that a wide variety of substrate materials could be used for ECM deposition without detriment to the bioactivity of the surface, expanding the use of MSC dECM technology for a wide variety of applications requiring different substrates.

## Data Availability Statement

The original contributions presented in the study are included in the article/supplementary material, further inquiries can be directed to the corresponding author/s.

## Ethics Statement

The studies involving human participants were reviewed and approved by the Human Research and Ethics Committee at the Women's Hospital, Melbourne, Australia (HREC#14/35). The patients/participants provided their written and informed consent to participate in this study.

## Author Contributions

MY: 60%, experimental design, data collection, and manuscript preparation. AO'C and BK: 10%, experimental design, and manuscript preparation. DH: 20%, experimental design, and manuscript preparation. All authors contributed to the article and approved the submitted version.

## Funding

This work was supported by the Victorian Medical Research Acceleration Fund (2019-Round 3) and an ARC Future Fellowship (FT190100280). We gratefully acknowledge the support of the University of Melbourne and an Australian Government Research Training Program Scholarship.

## Conflict of Interest

The authors declare that the research was conducted in the absence of any commercial or financial relationships that could be construed as a potential conflict of interest.

## Publisher's Note

All claims expressed in this article are solely those of the authors and do not necessarily represent those of their affiliated organizations, or those of the publisher, the editors and the reviewers. Any product that may be evaluated in this article, or claim that may be made by its manufacturer, is not guaranteed or endorsed by the publisher.
